# *Saccharomyces cerevisiae* β-glucan improves the response of trained macrophages to severe *P. aeruginosa* infections

**DOI:** 10.1007/s00011-024-01898-1

**Published:** 2024-06-08

**Authors:** Marta Ciszek-Lenda, Bernadeta Nowak, Grzegorz Majka, Maciej Suski, Maria Walczewska, Angelika Fedor, Edyta Golińska, Sabina Górska, Andrzej Gamian, Rafał Olszanecki, Magdalena Strus, Janusz Marcinkiewicz

**Affiliations:** 1 Department of Immunology, Faculty of Medicine, Jagiellonian University Medical College, Czysta 18, Krakow, 31-121 Poland; 2 Department of Pharmacology, Faculty of Medicine, Jagiellonian University Medical College, Grzegorzecka 16, Krakow, 31-53 Poland; 3 Department of Microbiology, Faculty of Medicine, Jagiellonian University Medical College, Czysta 18, Krakow, 31-121 Poland; 4 Hirszfeld Institute of Immunology and Experimental Therapy, Department of Microbiology, Laboratory of Microbiome Immunobiology, Polish Academy of Sciences, Weigla 12, Wroclaw, 53-114 Poland; 5 Hirszfeld Institute of Immunology and Experimental Therapy, Department of Immunology of Infectious Diseases, Laboratory of Medical Microbiology, Polish Academy of Sciences, Weigla 12, Wroclaw, 53-114 Poland; 6 University of Agriculture, University Centre of Veterinary Medicine, Mickiewicza 24/28, Krakow, 30- 059 Poland

**Keywords:** Trained macrophages, Inflammation, Biofilm *P. Aeruginosa*, *Saccharomyces cerevisiae* β-glucan

## Abstract

**Objective P. Aeruginosa:**

(PA), the major pathogen of lung cystic fibrosis (CF), polarizes macrophages into hyperinflammatory tissue damaging phenotype. The main aim of this study was to verify whether training of macrophages with β-glucan might improve their response to *P. aeruginosa* infections.

**Methods:**

To perform this task C57BL/6 mice sensitive to infections with *P. aeruginosa* were used. Peritoneal macrophages were trained with *Saccharomyces cerevisiae* β-glucan and exposed to PA57, the strong biofilm-forming bacterial strain isolated from the patient with severe lung CF. The release of cytokines and the expression of macrophage phenotypic markers were measured. A quantitative proteomic approach was used for the characterization of proteome-wide changes in macrophages. The effect of in vivo β-glucan-trained macrophages in the air pouch model of PA57 infection was investigated. In all experiments the effect of trained and naïve macrophages was compared.

**Results:**

Trained macrophages acquired a specific phenotype with mixed pro-inflammatory and pro-resolution characteristics, however they retained anti-bacterial properties. Most importantly, transfer of trained macrophages into infected air pouches markedly ameliorated the course of infection. PA57 bacterial growth and formation of biofilm were significantly suppressed. The level of serum amyloid A (SAA), a systemic inflammation biomarker, was reduced.

**Conclusions:**

Training of murine macrophages with *S. cerevisiae* β-glucan improved macrophage defense properties along with inhibition of secretion of some detrimental inflammatory agents. We suggest that training of macrophages with such β-glucans might be a new therapeutic strategy in *P. aeruginosa* biofilm infections, including CF, to promote eradication of pathogens and resolution of inflammation.

**Supplementary Information:**

The online version contains supplementary material available at 10.1007/s00011-024-01898-1.

## Introduction

Biofilm forming species of *P. aeruginosa* (PA) are major pathogens of chronic respiratory tract infections including lung cystic fibrosis (CF) [1]. In such infections accompanying chronic inflammation and tissue injury is caused by both bacterial virulence factors and cytotoxic agents (elastase, TNF-α, NO, ROS) released from inflammatory cells (neutrophils and macrophages) [2]. On the other hand, M-2 macrophages seem to be the dominant cells responsible for the resolution of inflammatory response [3]. Previously we have shown that components of *P. aeruginosa* biofilm matrix, especially exopolysaccharides (EPS), polarize macrophages towards M-1 hyperinflammatory cells. We have proposed a new term for macrophages polarized in the presence of biofilm matrix components as biofilm-associated macrophages (BAMs) [4]. These cells exhibit secretory properties similar to M1 macrophages characterized by increased levels of TNF-α, IL-6, IL-12p40, PGE_2_ and NO as well as by upregulation of NOS2 (inducible) and COX-2 with no changes in arginase-1 expression. Importantly, thusly polarized macrophages, in spite of strong antibacterial properties, may have a detrimental effect on the course of chronic inflammatory infections [5].

These findings suggest that training of naïve macrophages, prior to the contact with pathogens that protect from the polarization into BAMs may be a new therapeutic strategy in chronic *P. aeruginosa* infections, including lung CF. We addressed the issue whether it might be achieved by treatment of macrophages with β-glucan, the primary inducer of trained immunity [6].

Innate immune cells, such as monocytes/macrophages can develop M1-like inflammatory phenotype and exacerbate immunologic response following brief exposure to various inducers. It leads to an altered response towards a second challenge [7]. Such phenomenon, known as trained immunity, has been demonstrated after using β-glucans derived from Candida species [8]. Fungi of *Candida* genus despite being a normal part of the gut microbiome, can cause infections if they overgrow and disseminate [9]. Therefore, it has been reasonable to replace proinflammatory β-glucan derived from potential pathogens with anti-inflammatory β-glucan from probiotic *S. cerevisiae* [10, 11].

The aim of this study was to evaluate a phenotype of murine macrophages in vitro trained with β-glucan (BG) from *S. cerevisiae* before and after the exposure to *P. aeruginosa* (PA57). Moreover, we have investigated defensive/immunomodulatory properties of the in vivo trained macrophages transferred into mice infected with PA57.

In order to perform this task we used peritoneal exudate cells from C57BL/6 mice previously administered intraperitoneally with thioglycolate. This strain of mice have been chosen due to its sensitivity to *P. aeruginosa* infections [12]. β-glucan (BG) was derived from *S. cerevisiae* as a trained immunity inducer and the response to bacterial strain PA57 was being evaluated. PA57 is a high biofilm-forming strain isolated from a patient with severe lung CF [13]. Macrophages were trained in accordance with previous reports [14]. In the in vitro experimental system, the secretory cytokine response, phagocytic activity, bactericidal potential as well as quantitative proteomics analysis were performed to compare the phenotype of trained and untrained macrophages.

In the in vivo study we focused on elucidating the effect of macrophages in the air pouch model employing *P. aeruginosa* (PA57) infection [15]. Herein, we compared the impact of transfer of in vivo trained macrophages with that of non-trained cells on the efficiency of bacterial clearance (locally at a site of infection and spleen), infiltration of inflammatory cells, biofilm formation within the air pouch, as well as on the serum level of major markers of systemic inflammation.

## Materials and methods

### Reagents

β-glucan isolated from *Saccharomyces cerevisiae*, a major structural element of the yeast cell wall having a β1,3-glucan linear structure with a small number of β1,6-glucan branches.(Merck KGaA, Darmstadt, Germany).

### Bacteria

All experiments in this study were performed using *P. aeruginosa* strain coded as PA57. Bacteria were isolated from sputum of the patient during an exacerbation of the advanced stage of lung CF. PA57 had strong biofilm production capacity, and was isolated from the patient with a severe form of the disease [13]. Isolated bacteria were cultured in tryptic soy broth (TSB, Oxoid/ThermoFisher Scientific, Fremont, CA, USA) for 72 h at 37 °C under aerobic conditions. After cultivation, bacteria were centrifuged for 10 min at 500 g and washed with 10 mL of phosphate buffered saline (PBS, pH 7.4, Sigma-Aldrich, Steinheim, Germany) then used for in vivo tests. For in vitro tests with immune cells bacteria were killed (see below).

### Killing *P. Aeruginosa* bacterial cells

Bacteria pellets originating from 72 h cultures were treated thrice with high temperature (121 °C) at 0.3 bars in the ASVE-ELMI ESS-207 SMS steam sterilizer and in that form were used to stimulate immune cells (see below). The follow-up bacteria culture was verified to be sterile.

### Mice

Inbred C57BL/6 mice (8–12 weeks of age, 18–22 g) were maintained at the Animal Breeding Unit of the Department of Immunology of Jagiellonian University Medical College. All mice were held in standard caging conditions with water and standard diet ad libitum.

### Cell isolation

For in vitro assays, peritoneal mouse exudate cells were induced by an intraperitoneal injection of 1.5 mL of 3% thioglycolate (Sigma-Aldrich). After 96 h (macrophages) mice were euthanized by overdosing of isoflurane vapors (Abbott Laboratories, Chicago, IL, USA) and cervical dislocation was performed. Cells were then collected by washing out the peritoneal cavity with 5 mL of PBS (Lonza, Verviers, Belgium) containing 5 U heparin/mL (Polfa, Warsaw, Poland). Cells were centrifuged, and red blood cells were lysed. Osmolarity was restored by addition of PBS. At least three mice were used as donors of peritoneal exudate cells for each experiment. For in vivo tests, mice were injected intraperitoneally with 1 mg BG suspended in 1 mL PBS on days − 7, -4, and − 1 BG treated mice were euthanized by overdosing of isoflurane vapors (Abbott Laboratories) one day after last injection of BG. Cells from BG-treated and from naïve mice (donor mice) were collected from peritoneal cavity as described above. After washing cells were suspended in PBS at the concentration of 1 × 10^7^ cells/mL and injected into air pouches at the volume of 0.1mL i.e. 1 × 10^6^ cells/pouch/mouse.

### Cell viability

Cell viability was monitored by means of LDH activity (lactate dehydrogenase) using LDH assay kit (Thermo Fisher Scientific, Rockford, IL, USA) according to manufacturer’s instruction. The viability of phagocytes was controlled in all experimental systems to avoid cytotoxic effect of the tested agents.

### Cell culture and treatment

Macrophages were cultured in 24-well flat-bottom cell culture plates at 5 × 10^5^/well in IMDM medium (Lonza) supplemented with 5% fetal bovine serum (FBS; Lonza), 2 mM stable L-glutamine (Lonza), and 50 mg/mL gentamicin (KRKA, Warsaw Poland) at 37 °C in an atmosphere of 5% CO_2_. To train macrophages, cells were incubated for 24 h with 10 µg/mL of BG and then exposed to selected bacteria (20:1 bacteria per cell). After another 24 h of stimulation, culture supernatants were collected and frozen at − 80 °C until use.

### Cytokine measurement

Cytokine levels in cell culture supernatants were measured by sandwich ELISA. Microtiter plates (Costar EIA/RIA plates, Corning) were coated with a cytokine-specific antibody. Expression levels of IL-6 and IL-10 were measured according to the manufacturer’s instructions (OptEIA Sets, BD Biosciences, San Diego, CA, USA). TNF-α level was measured according to the manufacturer’s instructions (ELISA uncoated kits, Invitrogen, Waltham, MA, USA). In all cases, 10% FBS in PBS was used as a blocking solution. TMB substrate solution (Invitrogen) was used to develop a colorimetric reaction, which was stopped with 2 M sulfuric acid. Optical density was measured at 450 (570) nm using a microtiter plate reader (PowerWaveX, Bio-Tek Instruments, Winooski, VT, USA).

#### Prostaglandin E2 (PGE2) immunoassay

PGE_2_ concentration in cell supernatants was determined by a PGE2 high-sensitivity ELISA kit (Enzo Life Sciences, Farmingdale, NY, USA), according to the manufacturer’s protocol.

### SAA determination

SAA concentration in mouse serum was determined by SAA ELISA kit (Invitrogen), according to the manufacturer’s protocol.

### Nitric oxide (NO) determination

NO levels in culture supernatants of macrophages were quantified by the accumulation of nitrite as a stable end product, according to a modified Griess method [16]. Cell culture supernatant (100 µL) was mixed with 14 mM 4,4′-diamino-diphenylsulphone (Dapsone, Sigma-Aldrich) in 2 M HCl (50 µL) and 0.1% N-1-naphtylenediamine dihydrochloride (50 µL) in deionized water. Absorbance of the tested culture supernatants at 550 nm was compared with a sodium nitrate standard (NaNO_2_) curve.

### Proteomics

Sample preparation, protein digestion and SWATH mass spectrometry analysis were described in detail in our previous report [17]. Briefly, macrophages were lysed in 2% SDS, 50 mM DTT in 0.1 M Tris-HCl pH 7.6 and digested to peptides with a use of filter-aided sample preparation (FASP) protocol [18] with LysC-trypsin mix (Thermo Scientific, Waltham, MA, USA) at the enzyme to protein ratio 1:50. After digestion the peptide yields were determined by WF-assay [19] and the aliquots containing equal amount of total peptides were desalted on 96-Well MiniSpin C18 columns (Harvard Apparatus, Holliston, MA, USA). Peptides (1 µg) were injected onto a nanoEase M/Z Peptide BEH C18 75 μm i.d. × 25 cm column (Waters, Milford, MA, USA) via a trap column nanoEase M/Z Symmetry C18 180 μm i.d. × 2 cm column (Waters). Samples analysed in SWATH acquisition mode were separated using a 63 min 1–40% B phase linear gradient (A phase − 0.1% FA; B phase − 80% ACN and 0.1% FA) at a flow rate of 300 nL/min by UltiMate 3000 HPLC system (Thermo Scientific, Waltham, MA, USA) and applied to a TripleTOF 6600+ (Sciex, Framingham, MA, USA) mass spectrometer. The main working nano-electrospray ion source (Optiflow, Sciex, Framingham, MA, USA) parameters were as follows: ion spray voltage 3.2 kV, interface heater temperature (IHT) 200 °C, ion source gas 1 (GS1) 10 and curtain gas (CUR) 25. Spectra were collected in full scan mode (400–1250 Da), followed by one hundred SWATH MS/MS scans using a variable precursor isolation window approach, with m/z windows ranging from 6 to 90 Da.

Project-specific library (preparation described in detail in [17]) was used to analyse the SWATH data in Spectronaut 16 (Biognosys, Schlieren, Switzerland). Data were filtered by 1% FDR on peptide and protein level, while quantitation and interference correction were done on the MS2 level. Protein grouping was performed based on the ID picker algorithm [20]. Protein quantities were calculated by averaging the respective peptide intensities, while the latter were obtained as mean precursor quantities. The protein coefficients of variation (CVs) were calculated based on the summed intensities of their respective peptides. Data were normalized by global regression strategy, while statistical testing for differential protein abundance was done using t-tests with multiple testing correction after Storey [21]. Statistically significant differences (q value < 0.05) with quantitative cut-off for absolute 1.5 fold change were considered as differentially regulated. The LC-MS data, library and Spectronaut project have been deposited to the ProteomeXchange Consortium via the PRIDE partner repository [22] with the dataset identifier PXD036521. Functional grouping and pathway annotations were performed using ClueGO (PMID: 19,237,447) under the Cytoscape 3.8.2 software environment (PMID: 14,597,658). CORUM-3.0 (release 03.09.2018), KEGG (release 17.02.2020), REACTOME (release 17.02.2020) and WikiPathways (release 17.02.2020) pathways were used in the analysis. Enrichment results were validated by enrichment/depletion two-sided geometric statistical test with Bonferroni step down as p value correction method. Details of the enrichment analyses are summarized in Supplementary Table S3. Minimum and maximum GO levels were set as 1 and 4, respectively, with the cluster criteria of minimum 5 genes constituting a minimum of 2% of the GO term. Kappa score threshold was set as 0.4.

### Phagocytosis and bacterial killing assay

Macrophages were incubated for 90 min. at 37 °C with 3.2 × 10^6^ CFU of tritium-labeled PA57 in 0.1 mL RPMI with 5% FCS (without antibiotics) [23]. Following the incubation the cells were washed three times with PBS (140 µL) and lysed using 100 µl of 0.5% SDS in water. Lysates aliquots (90 µL) were transferred to 96-well white, clear bottom plates (PerkinElmer, Waltham, MA, USA) and subjected to overnight evaporation at 50 °C. Subsequently the wells were filled with 150 µL of Opti-Fluor scintillator (Perkin Elmer) and after 40 min. incubation the radioactive signal was measured in a microplate scintillation counter (MicroBeta TriLux, Wallac Turku Finland). The populations of viable intracellular PA57 bacteria were assessed using the gentamycin protection assay. After 60 min. incubation with PA57, unbound bacterial cells were washed out and RPMI with 5% FCS and 0.4 mg/mL gentamycin was added for another round of incubation at 37 °C (30 min.). Afterwards, the cells were washed once with PBS and either lysed immediately in 0.1 mL of 0.1% Triton X-100 (Sigma-Aldrich) in PBS or after 2 h incubation in RPMI with 5% without gentamycin. Cells lysates were sonicated for 5 min. and serial dilutions were plated on blood agar plates. CFU of original samples was established from the colonies counted after overnight culture at 37 °C.

### The air-pouch model of infection

To create air-pouches 5 mL of sterile air was injected subcutaneously onto thoracic region of the back of each mouse (Fig. 6). Then, 3 days later, to maintain the pouch, each pouch was re-inflated with 5 mL of sterile air and left for 1 more day. The sterile air was obtained by filtration through Millipore filter (0.22 μm) directly into a sterile 10 mL syringe in the laminar flow station. Bacteria PA57, 1 × 10^7^ CFU suspended in 0.1 mL PBS were injected into each air pouch. Infected mice (recipient mice) were divided into 3 groups (*n* = 3). Immediately after infection mice received intrapouch injection of 0.1 mL volume of (group A) PBS, or (group B) 1 × 10^6^ cells isolated from peritoneal cavity of naïve mice or (group C) 1 × 10^6^ cells isolated from peritoneal cavity of mice trained with BG (as described above) 6 days after infection mice were euthanized. An air pouch lavage was performed by repeated injection/aspiration of 1 mL PBS. The lavage was examined for bacterial load and the presence and characteristics of host immune cells. The cellular fraction of the air pouch lavage was obtained by centrifugation, (300 g, 5 min). Cells were washed, stained with fluorochrome labeled antibodies against cell type-specific markers and analyzed analysed by flow cytometry. Air pouches were cut-opened and examined for the presence of biofilm. Additionally, blood and spleens were collected from infected mice. CFU count in these tissues and in the air pouch lavage was performed after 24 h culture on Columbia agar plates (Oxoid/ThermoFisher Scientific).

### Flow cytometry analysis

Cells, i.e. peritoneal cavity cells used as donor cells (naïve and BG-trained) injected into air pouches, and cells harvested from air pouches at day 6 after infection were washed in washing buffer (PBS containing 2% FBS) and stained for 30 min. at 4 °C in 50 µL staining buffer (PBS containing 2% FBS and anti-mouse CD16/CD32 antibody (Fc block) (BD Biosciences) with the combination of the following cell surface marker antibodies conjugated with fluorochromes: α-CD11b-FITC (BD Biosciences), α -Ly6C-PE, α-Ly6G-PE (both from BioLegend, San Diego, CA, USA), PE conjugated α-mouse neutrophils 4/7 antibody (Abcam), α-I-Ab-PE, α-CD80-PE, α-CD86-PE (all from BD Biosciences), α-TLR2-PE (BioLegend). For intracellular staining following antibodies: α-CD68-APC (BioLegend) or α-CD68-PE (BD Biosciences), α-CD206-APC (BioLegend), α-Arginase1-APC (Invitrogen) were used. Cells were permeabilized with reagents provided in Cytofix/Cytoperm Fixation/Permeablization Kit (BD Biosciences) according to the manufacturer instruction. Isotype control antibodies (rat IgG2b-FITC, rat IgG2a-PE, rat IgG2a-APC) were used to set positivity threshold. To assess phagocytic capability of naïve vs. BG-treated cells suspended in saline cells were incubated (2 h, 37 °C) with pHrodo Zymosan Green Bioparticles (Thermo Fisher Scientific). Phagocytosis was stopped with ice-cold buffer (PBS) and then cells were washed and stained with PE-conjugated anti-CD11b monoclonal antibody (BD Biosciences). The stained cells were analyzed on FACSCalibur using CellQuest Pro Software. The data were analyzed in FlowJo_v10.9 software.

### Statistical analysis

Statistical significance of differences between groups was analysed using one-way ANOVA or Student’s t-test Results are expressed as mean ± SEM values. A P value < 0.05 was considered statistically significant. Analysis was performed using Graphpad Prism v. 5.01 (GraphPad Software, Inc. San Diego, CA, USA). For proteomics statistical analysis see above in the Methods. The exact statistical analysis and post hoc comparison are named in the relevant figure’s legend.

## Results

### Impact of β-glucan training on the macrophage production of cytokines, NO and PGE_2_ in vitro

To evaluate the effect of macrophage training on secretory properties we used *S. cerevisiae* BG. The effective stimulatory concentration of BG (10 µg/mL) was established in preliminary tests (Supplemental Fig. 1). At this concentration of BG, macrophages showed proinflammatory profile of the tested mediators, but with a very low level of IL-6 as compared with that observed after the stimulation with PA57. Moreover, BG did not stimulate the production of NO (data not shown).

**Fig. 1:**
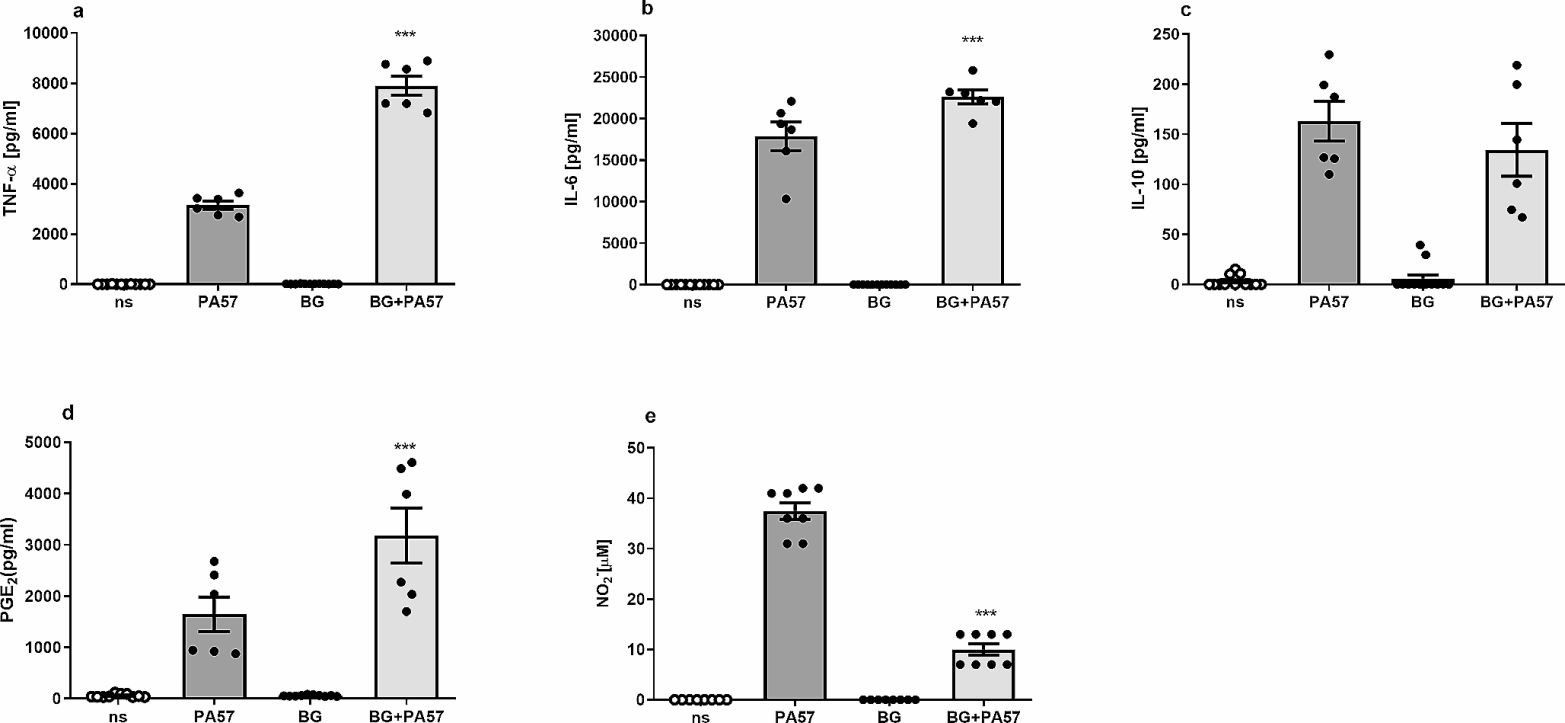
Secretory properties of BG-trained macrophages exposed to PA57 bacteria cells. Levels of TNF-α (**a**), IL-6 (**b**), IL-10 (**c**), PGE2 (**d**) and NO (**e**) were analysed by ELISA or Griess method, respectively, in supernatants collected 24 h after the restimulation of macrophages with PA57. Data are mean ± SEM values of three independent experiments. Each group was run in duplicates or triplicates. ***p < 0.001 BG + PA57vs PA57, Student’s t-test

Furthermore, macrophages were primed with BG for 24 h and then after exchange of the culture medium were exposed to killed whole PA57 for another 24 h. The results showed that BG training increased production of TNF-α, IL-6 and PGE_2_, (Fig. 1a, b, d) but reduced secretion of IL-10 (Fig. 1c) by macrophages restimulated with PA57. This suggests pro-inflammatory properties of these macrophages in the interaction with PA57. Interestingly, training with BG significantly lowers the level of NO upon PA57 re-stimulation of macrophages (Fig. 1e).

### Impact of β-glucan on phagocytic and bacteria killing properties of macrophages

**Fig. 2:**
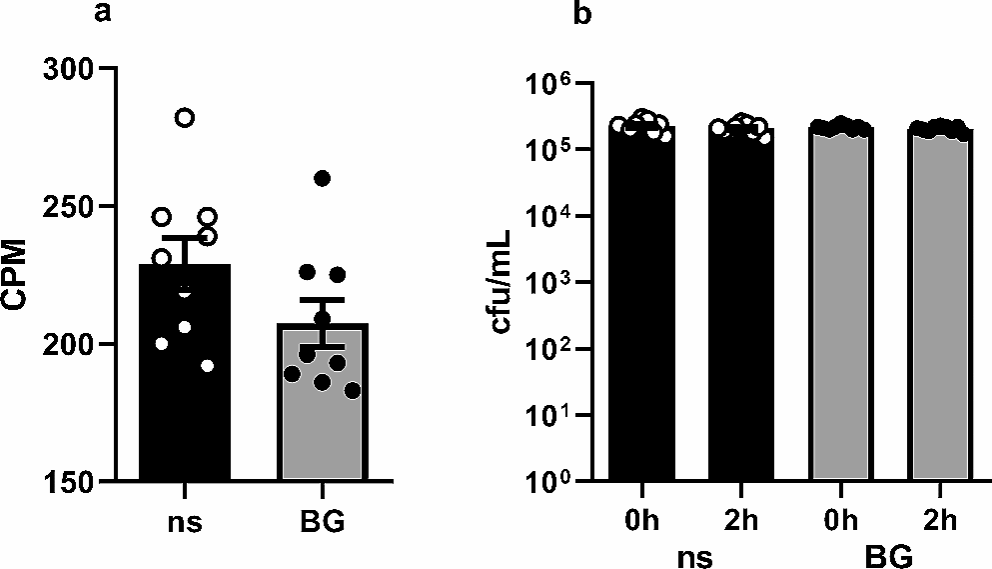
Effect of β-glucan training on phagocytic and intracellular killing activity of macrophages (**a**). Phagocytic activity was measured by assessing radioactivity of macrophages upon incubation with tritium-labelled PA57 bacteria, counts per minute (CPM) read by microplate scintillation counter (**b**). Populations of intracellular bacteria were assessed by plating the lysates of cells immediately upon incubation of macrophages with PA57 and 2 h later for control and BG pre-treated macrophages. Data are mean ± SEM values of three independent experiments. Each group was run in triplicates. ns-not stimulated. Student’s t-test was used to compare phagocytic activity (**a**) and Wilcoxon matched-pairs signed rank test was used to compare the bactericidal activity (**b**) of the macrophages

In order to assess the influence of β-glucan training on phagocytic potential of macrophages two assays have been used. Measurement of 3 H-thymidine incorporation has implicated that the level of PA57 internalization remains the same in both the control macrophages as well as those pre-treated with BG pre-treated macrophages in comparison to control group (Fig. 2a). Similarly, no changes were observed in the intracellular killing activity measured in the gentamycin-protection assay (Fig. 2b). It seems that both phagocytosis and the bactericidal potential of the trained macrophages towards the relevant *P. aeruginosa* strain is not altered upon training with the used BG.

### Impact of PA57 on the proteome of β-glucan trained macrophages versus naïve macrophages

**Fig. 3:**
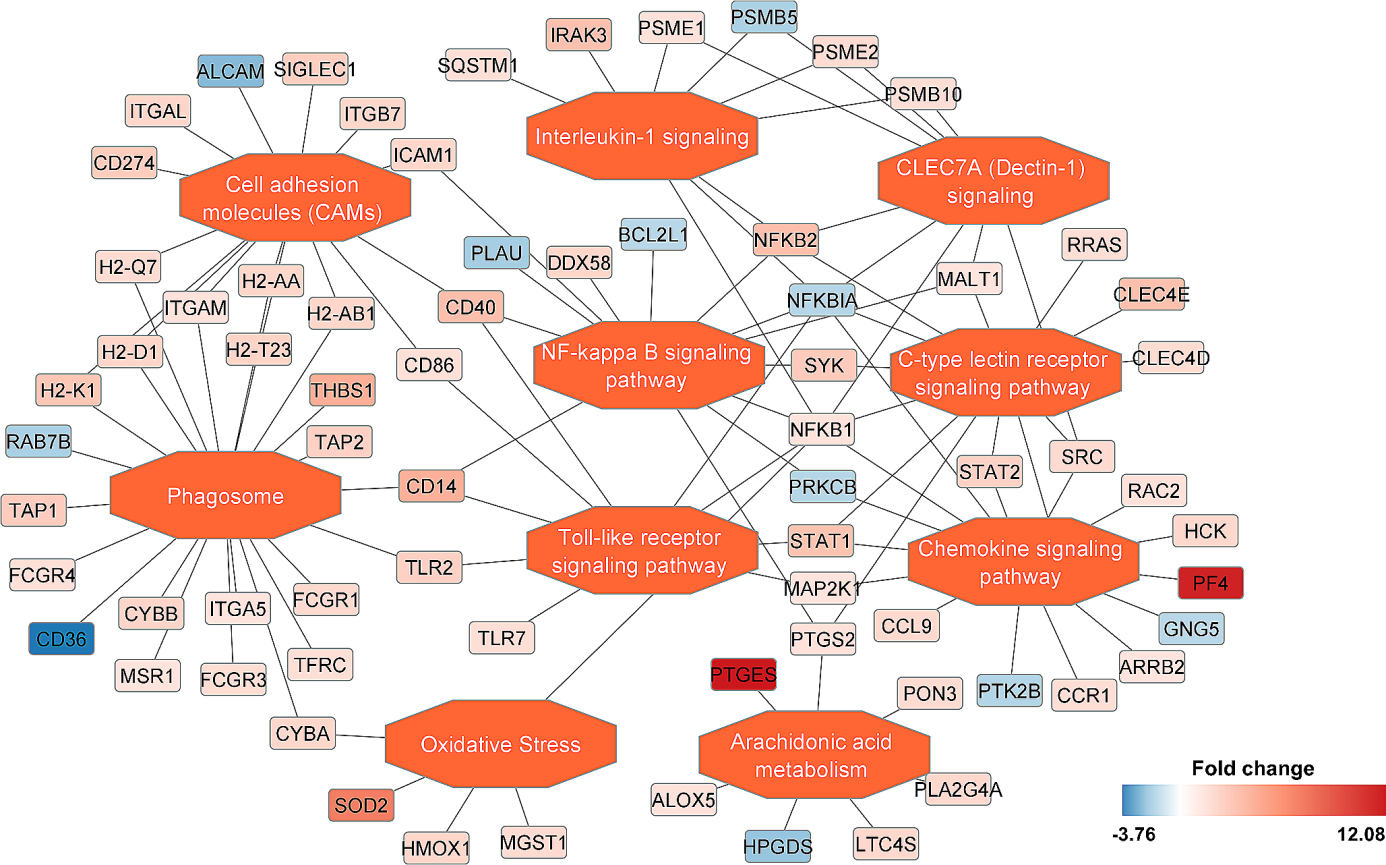
Functional grouping and pathway annotations of the differentially regulated proteins in response to BG stimulation. Pathway enrichment points to the key processes in which the regulated proteins were involved, including induction of dectin-1 signalling, NF-κB activation with downstream chemokine and interleukin-1 signalling pathways, as well as proteins related to the assembly and activity of phagosomes as the main features. Additionally BG stimulation upregulates proteins with antioxidant properties (i.a. mitochondrial superoxide dismutase 2 and hemoxygenase-1) and those involved in the metabolism of prostaglandins

**Fig. 4:**
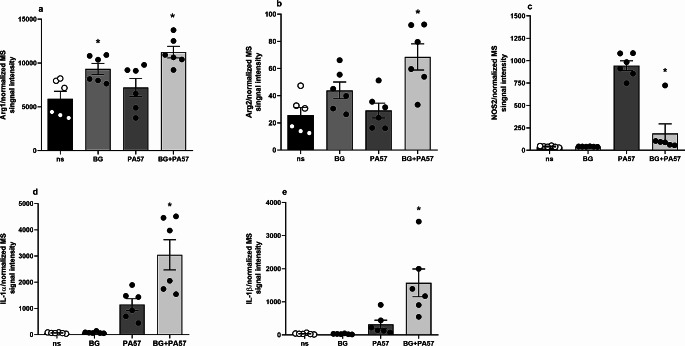
Differential expression changes of selected M1 and M2 macrophage protein markers. Proteomic evaluation of quantitative changes in classical macrophage phenotype markers indicates that BG training results in arginases induction (**a**, **b**), which was further increased in stimulated cells. On the contrary, M1 associated interleukins and iNOS (**c**) were not affected by BG training, but when activated, they exhibited differential abundance changes: macrophages pre-treated with BG responded with stronger induction of interleukin-1 but repressed iNOS level (**b**, **c**, **d**). Data are mean ± SEM values of 6 independent experiments. ns-not stimulated. *p < 0.05 ns vs. BG and PA57 vs. BG + PA57, statistical analysis was performed with Student’s t-test

**Fig. 5:**
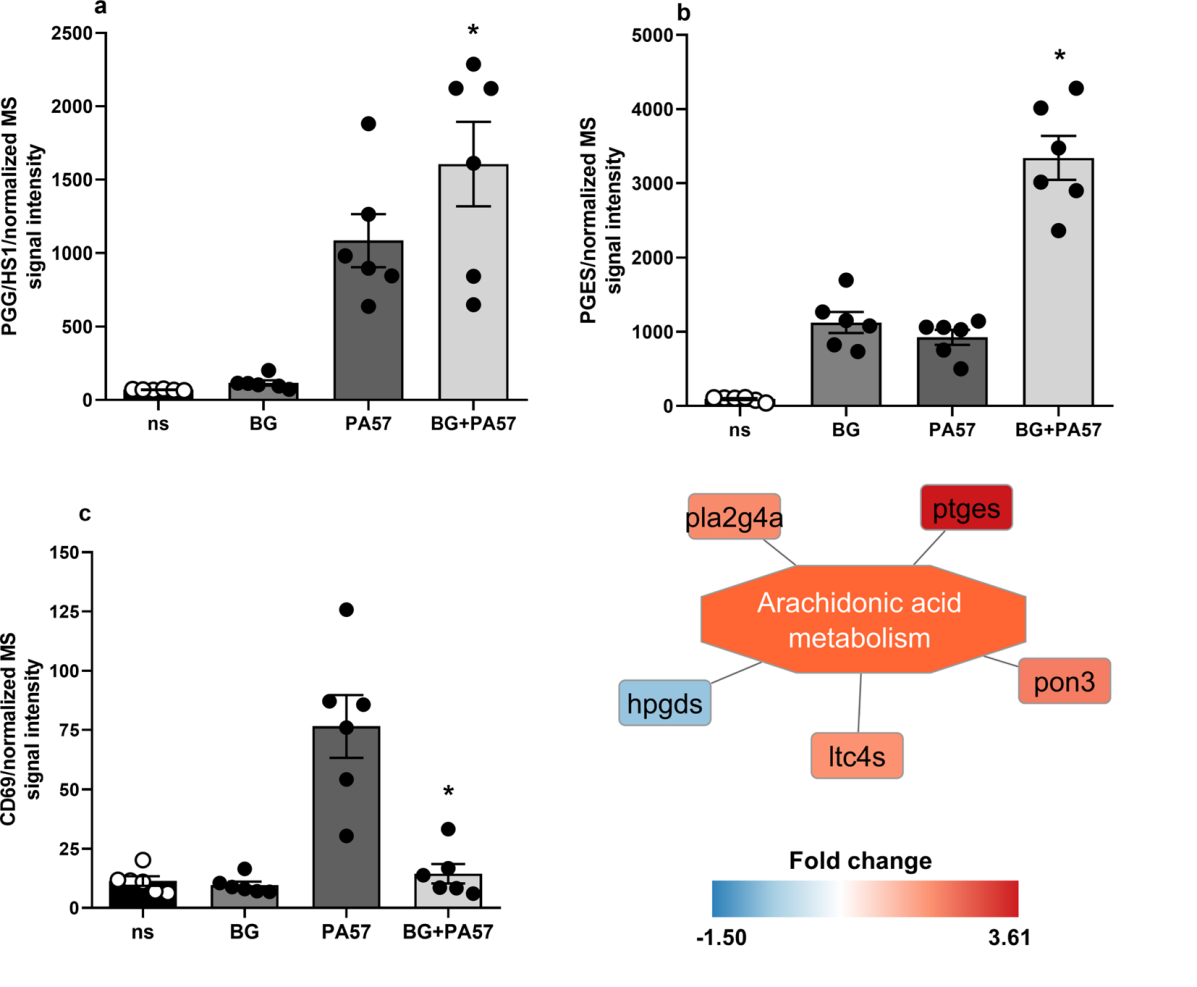
Functional differences in eicosanoids metabolism in trained macrophages. Differentially regulated proteins in BG pre-treated macrophages stimulated with PA57 bacteria functionally aggregate i.a. in the regulation of arachidonic acid metabolism, showing upregulation of components of the PGE2 biosynthesis pathway. The latter is further supported by the inverse changes of PGES and CD69 in the macrophage proteome. Data are mean ± SEM values of 6 independent experiments. ns-not stimulated. *p < 0.05 PA57 vs. BG + PA57, statistical analysis was performed with Student’s t-test

DIA quantitative proteome measurements revealed that 356 proteins were differentially regulated in macrophages trained with BG as compared to the unstimulated control group (Suppl. Table S1). As expected, most of the identified proteins were functionally related to the activation and propagation of the immune response and induction of inflammation with a numerous cytokine and chemokine signaling pathways regulated upon BG action (Fig. 3). Our analysis has shown that trained macrophages acquire a specific phenotype with mixed pro-inflammatory and pro-resolution characteristics. Induction of classic markers and signaling pathways of the M1-like phenotype (including induced dectin-1 and subsequent NF-κB, signaling, other lectin and TLR receptors activation, differences in adhesion molecules and proteins involved in phagocytosis) was accompanied by quantitative changes in proteins associated with M2-like polarization (including induction of arginase 1 and 2, antileukoproteinase, PAI-2, or superoxide dismutase) (Fig. 4a, b). Additionally, BG did not induce classic M1 markers such as IL-1α, IL-1β or iNOS in macrophages. Interestingly, BG strongly enhanced IL-1α and IL-1β synthesis with concomitant suppression of iNOS expression in trained macrophages in response to subsequent activation with PA57 bacteria (Fig. 4c, d, e, Suppl. Table S2). The latter was accompanied by induction of protein components of mitochondrial electron transport chain (mainly of Complex I) and phagosome (including TRL-2 and CD14) (Fig. 3, Suppl. Figure 2). Proteomic analysis also confirmed the observed functional induction of PGE2 synthesis, proving that BG not only increased the concentration of prostaglandin E2 synthase (correlated with CD69 repression), but also enhanced its biosynthesis in macrophages stimulated with PA57 (Fig. 5a, b, c).

### Phenotype and function of in vivo BG trained cells

**Fig. 6:**
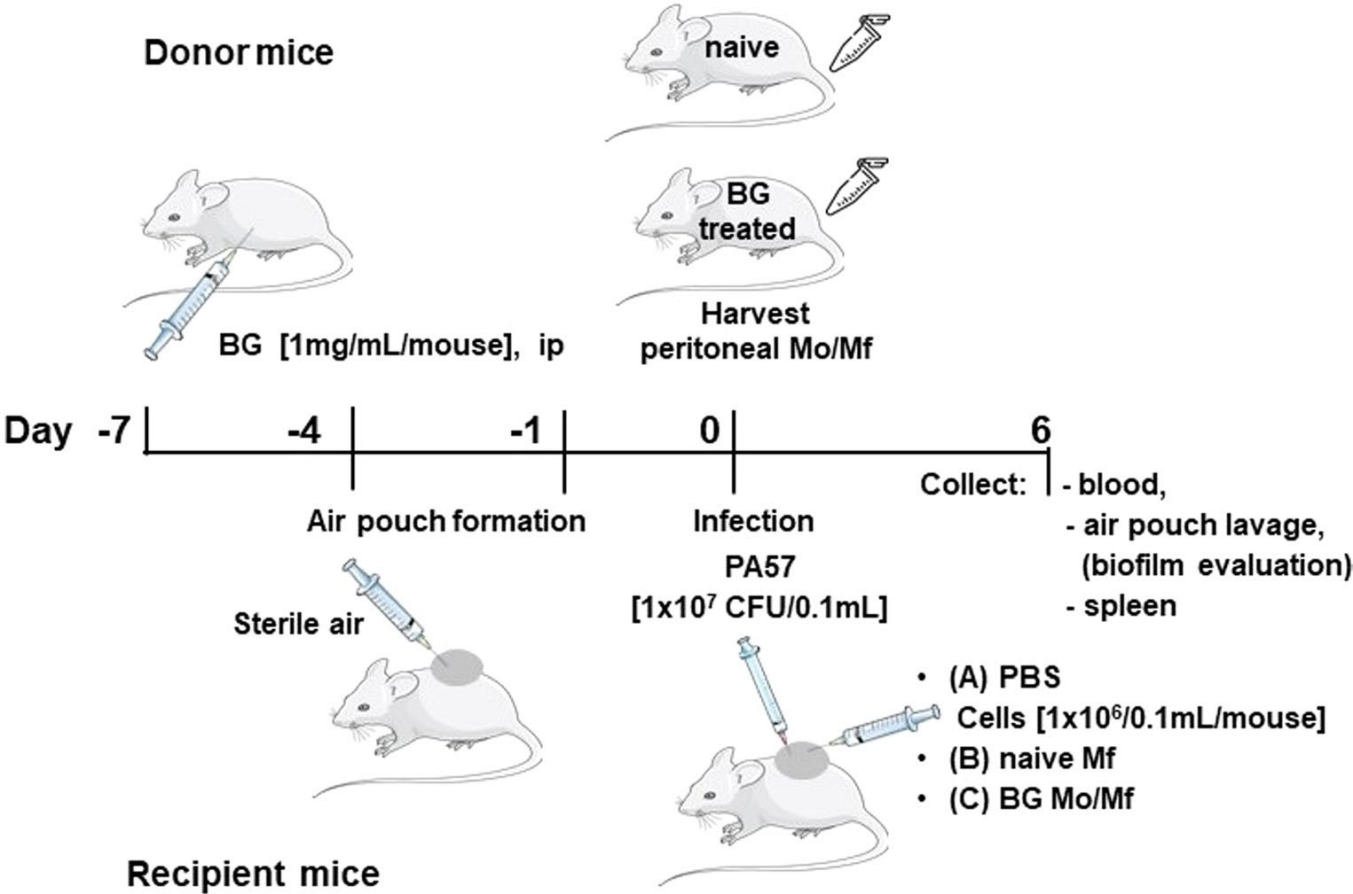
Scheme of the air-pouch experimental model

**Fig. 7:**
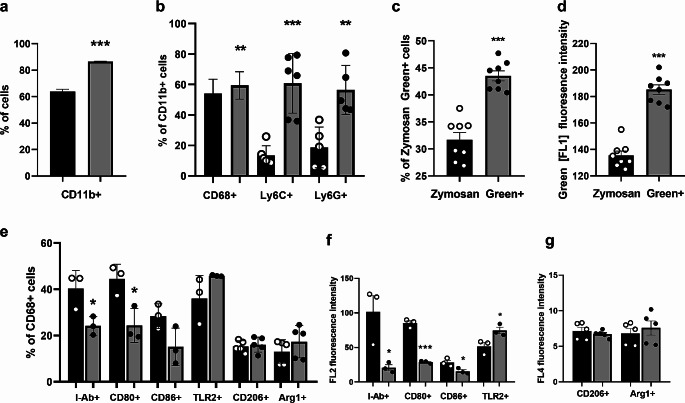
Characteristics of in vivo BG trained cells from mouse peritoneal cavity. Mice (C57BL/6, *n* = 9 in total i.e. *n* = 3 for each single experiment) were injected intraperitoneally with 1 mg β-glucan, on day 0, 3 and 6. On day 7 cells were harvested from the peritoneal cavity of BG treated mice. At the same time cells were harvested from naïve mice. Cells in the peritoneal cavity lavage were stained with monoclonal antibodies labelled with fluorescent dyes and analysed by flow cytometry. (**a**) The percentage (± SEM) of myeloid cells (CD11b^+^) in peritoneal cavity of naïve (black bars) and BG trained (gray bars) cells is shown. (**b**) Myeloid cells (CD11b^+^ gated) were examined for the occurrence (shown as percentage) of macrophages (CD68^+^ cells), monocytes (Ly6C^+^ cells) and granulocytes (Ly6G^+^ cells). To asses phagocytic capability of naïve vs. BG trained cells, they (cells) were incubated (2 h, 37 °C) with pHrodo Zymosan Green Bioparticles. (**c**) The percentage of Zymosan Green^+^ (phagocytosing) cells (CD11b^+^ gated) and (**d**) the amount of phagocytosed bioparticles, measured as the intensity of green fluorescence (FL1 geometric mean fluorescence intensity) is shown. (**e**) Myeloid cells (CD11b^+^ gated) were gated and characterised based on CD68 expression and the expression of functional markers: MHC class II (I-Ab), or CD80, or CD86, or TLR2, or CD206 or Arginase 1 (Arg1). The percentage of CD68^+^I-Ab^+^, CD68^+^CD80^+^, CD68^+^CD86^+^, CD68^+^TLR2^+^, CD68^+^CD206^+^ and CD68^+^Arg1^+^ of naïve (black bars) and BG trained (gray bars) cells is shown. (**f**) The level of expression of functional surface markers MHC class II (I-Ab), CD80, CD86, TLR2 is shown as geometric mean of FL2 fluorescence intensity and (**g**) the level of expression of intracellular markers: CD206 or Agr1 is shown as geometric mean of FL4 fluorescence intensity. The experiments were repeated 3-times. Data show the mean values from three independent experiments. Statistical analysis was performed with Student’s t-test, ***p < 0.001, **p < 0.01, *p < 0.05 naive vs. BG Mo/Mf

The cellular content of cells present in peritoneal cavity of C57BL/6 mice, naïve vs. BG treated, (donor mice, Fig. 6) was examined. The percentage of myeloid cells (CD11b^+^) in the peritoneal lavage of mice treated with BG was significantly higher compared to naïve mice (Fig. 7a). Additionally, among myeloid cells (CD11b^+^ gate, Suppl. Figure 3) the percentage of cells expressing CD68 (pan macrophage marker) and especially the percentage of cells expressing Ly6C (considered as monocytes) or Ly6G (granulocytes) was also significantly higher when mice were treated with BG in vivo (Fig. 7b). Furthermore, myeloid (CD11b^+^) cells from BG treated mice showed enhanced capability to phagocyte Zymosan Green Bioparticles. Not only the percentage of cells expressing Zymosan Green (Fig. 7c) but also the amount of phagocytosed bioparticles (referred to as the intensity of green fluorescence, expressed as geometric mean of green fluorescence intensity (MFI) (Fig. 7d) was significantly higher in case of BG trained cells (MFI = 185,3 ± 3,6) as compared to naïve cells (MFI = 133,5 ± 3,3).

Furthermore CD11b^+^ (myeloid) cells (gated) we examined for the expression of CD68^+^ (macrophages) and the expression of selected functional markers (MHC class II, CD80, CD86, TLR2) as well as intracellular expression of CD206 and arginase 1 (Arg1). As shown in Fig. 7e, after in vivo BG treatment the percentage of CD68^+^ cells expressing markers involved in antigen presentation (MHC class II, CD80) dropped down significantly. Reduced in number were also CD68^+^ cells expressing CD86. What more the level of expression of these markers (MHC class II, CD80, CD86) referred to as geometric mean of the fluorescence intensity (Fig. 7f) was also significantly reduced. While the percentage of CD68^+^ cells expressing TLR2 was enhanced, the difference between naïve and BG trained cells was not significant statistically (Fig. 7e). However the level of expression of TLR2 (FL2 MFI value) was significantly increased on BG trained cells (Fig. 7f). The percentage of CD68^+^ cells expressing CD206 or arginase 1 (Arg 1) did not change significantly after BG treatment (Fig. 7e). Similarly, no difference in the level of expression of CD206 or Arg1 was observed between naïve and BG trained cells (Fig. 7g). Flow cytometry data from one selected experiment are shown in Suppl. Figure 3.

### Air pouch model. biofilm formation and characteristics

#### Impact of β-glucan trained macrophages on innate immune defense against PA57 in mice

**Fig. 8:**
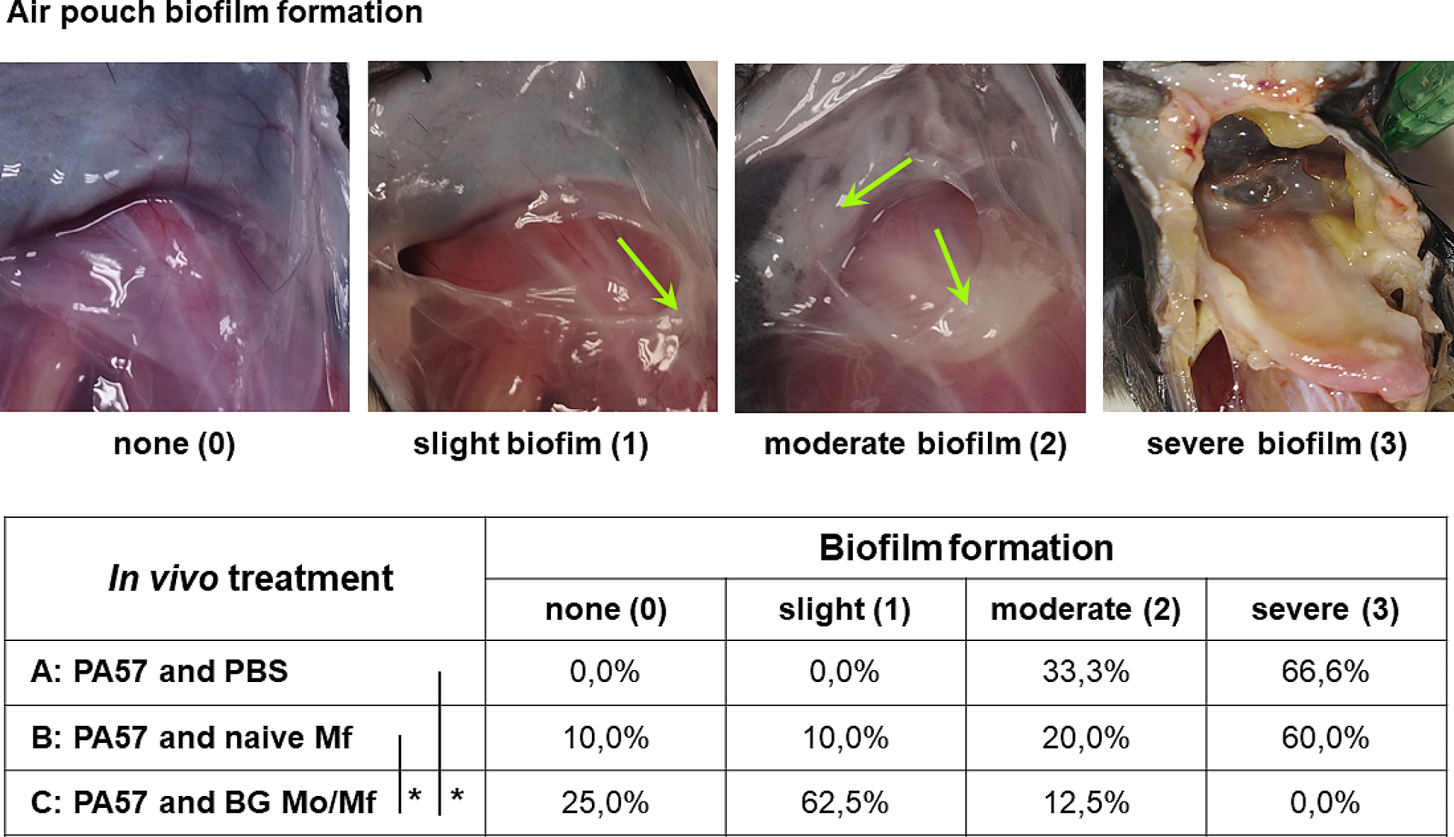
Biofilm formation in mouse air pouches. C57BL/6 mice (*n* = 24) were infected (day 0) with PA57 (1 × 10^7^/0.1mL) into air pouches formed on the back of the animals followed with the injection into air pouches of PBS (group A, *n* = 6) or cells harvested from peritoneal cavity (1 × 10^6^/0.1mL) of naive (group B, *n* = 10) or BG treated (group C, *n* = 8) C57BL/6 mice. On day 6 mice were sacrificed. Air pouches were cut opened and examined for the presence of biofilm. Based on the presence (or absence), abundance, visual characteristics and consistency of formed biofilm the following scale was introduced: (0) no biofilm formation (none), (1) slight biofilm (scarce, yellowish, flaky), (2) moderate biofilm (non-excessive, white or yellowish, mostly slime), (3) severe biofilm (abundant, slime, white or yellowish). The percentage of mice in each group with particular type of biofilm in their air pouches is shown. Statistical analysis was performed with Kruskal-Wallis test with post-hoc Dunn’s test, *p < 0.05 A vs. C and B vs. C

Mice were infected (day 0) with live PA57 bacteria (1 × 10^7^ CFU/0.1mL/air pouch). 6 days after infection air pouches were cut opened and examined for the presence of biofilm. Based on the presence (or absence), abundance, visual characteristics and consistency of formed biofilm the following scale was introduced: (0) no biofilm formation (none), (1) slight biofilm (scarce, yellowish, flaky), (2) moderate biofilm (non-excessive, white or yellowish, mostly slime), (3) severe biofilm (abundant, slime, white or yellowish) as shown in Fig. 8.

Mice infected with life bacteria PA57 (1 × 10^7^ CFU/0.1mL/air pouch) were given injection into air pouches of PBS (group A), naïve peritoneal cavity cells (group B) or BG trained cells (group C) (Fig. 6). Severe biofilm was observed in air pouches of most mice (66,6%) in the group A (PA57 infected, PBS) (Fig. 8). However, when naïve Mo/Mf (group B) were administered into air pouches immediately after infection with PA57, the formation of severe biofilm was slightly reduced (60% mice) and no severe biofilm was present in mice, which received BG trained Mo/Mf (group C). Although 75% of mice in group C had biofilm formed (12,5% mice had moderate biofilm and 62,5% mice had slight biofilm) in their air pouches indicating local presence of bacteria, the bacterial load in the air pouches was impressively reduced as compared to group A, as measured by CFU count in the air pouch lavage (Table 1). In the same time, the total number of host cells infiltrating the site of infection (present in the air pouch lavage) was greatly reduced, and the percentage of neutrophils (CD11b^+^4/7^+^ cells) among these cells also declined when BG trained Mo/Mf were injected into PA57 infected air pouches. Additionally, only 44,4% of mice in group C were positive for systemic (in the spleen) bacteria existence. In the same time, the systemic marker of acute inflammation (SAA in the blood) was significantly reduced (68 ± 26 µg/ml) as compared to mice, which did not receive Mo/Mf into infected air pouches (320 ± 130 µg/ml) (group A) (Table 1). More images of the biofilm formation in the opened air pouches of each group after PA57-infection and administration of naïve or BG-treated macrophages are included in the supplementary data. (Suppl. Figure 4).

**Table 1:** Air pouch model. The effect of macrophages on host defence against PA57

Parameters of host infection		In vivo treatment		
		(A) PA57 infected; PBS	(B) PA57 infected; Naïve Mf	(C) PA57 infected; BG Mo/Mf
Air pouch lavage	CFU	2,9 × 10^7^ ± 6,7 × 10^6^	4,4 × 10^6^ ± 1,9 × 10^6^	3,5 × 10^4^ ± 2,8 × 10^4^ *
	Number of host cells	5,8 × 10^7^ ± 7,4 × 10^6^	1,3 × 10^7^ ± 4,0 × 10^6^	3,0 × 10^6^ ± 6,1 × 10^5^ *
	Neutrophils	91,5 ± 2,1%	77,8 ± 7,4%	57,6 ± 2,7% *
Translocation of PA57 into the spleen [% of mice]		83,3%	57,1%	44,4%
Serum SAA [µg/ml]		320 ± 130	154 ± 57	68 ± 26

C57BL/6 mice (*n* = 27) infected (day 0) with life PA57 bacteria (1 × 10^7^ CFU/0.1mL) into air pouches formed on the back of the animal, were given single intrapouch injection of PBS (group A) or peritoneal cavity cells harvested from naïve (group B) or BG treated (group C) mice. On day 6 blood was collected (under light anaesthesia) and then mice were killed. Air pouches were cut opened and washed out with PBS. The content of bacteria (CFU) were examined in the air pouches lavage (local bacterial load) and in the spleen (systemic bacterial load). Host cells present in the air pouch lavage were counted and the total number of host infiltrating cells in the air pouches is shown. The percentage of neutrophils was determined by flow cytometry based on CD11b^+^4/7^+^ expression. The level of SAA in serum, which indicates systemic acute inflammation was measured by ELISA. The experiments were repeated 3-times. Data show the mean values from three experiments. Statistical analysis was performed with Student’s t-test *p < 0.05 naive vs. BG Mo/Mf

## Discussion

Macrophages might play either beneficial or detrimental role in infections and inflammatory diseases. Namely, in acute inflammation M1-proinflammatory macrophages support neutrophils in killing of pathogens [24]. After elimination of invaders and efferocytosis of apoptotic neutrophils, polarized M2-anti-inflammatory macrophages are involved in a resolution of inflammatory response and tissue repair. However, in some severe bacterial infections macrophages may be excessively stimulated to hyperinflammatory and tissue damaging activities [25]. Such situation has been described in interactions between biofilm forming *P. aeruginosa* strains and murine macrophages, named BAM (biofilm associated macrophages) [4]. More importantly, in lung cystic fibrosis (CF), macrophages infiltrating the airways infected by *P. aeruginosa* are not only non-effective phagocytes but they are also responsible for tissue injury, along with neutrophils [5].

Therefore, it was reasonable to try to improve macrophage response to PA57 (the bacterial strain isolated from the patient with severe lung CF) by generating a memory in innate immune cells, termed trained immunity. Notably, properly trained macrophages, *via* priming with various agents that epigenetically change their functional immune status, react to a subsequent contact with pathogens faster and more effectively than the naïve cells [26]. β-glucan from various Candida species is the most common training agent used to promote M1 macrophage polarization. This type of BG induced protective trained immunity with strong bactericidal properties of macrophages associated with high generation of NO, IL-1 and TNF-α [27, 28]. However, such trained macrophages might still be responsible for hyperinflammatory response and tissue injury.

On the other hand, macrophages trained with various β-glucans from *S. cerevisiae* species of yeast might show either pro- or anti-inflammatory properties when they are exposed to a secondary microbial stimulus [10, 11]. Importantly, Rizzetto et al. have reported that the major training effect of *S. cerevisiae* derived cell wall component was attributable to chitin rather than β-glucan. The effect was *S. cerevisiae* strain specific. Importantly, the trained cells were restimulated with various microbes, such as *S. aureus, E.coli or C. albicans*, but not with any biofilm-forming *P. aeruginosa* strain [29]. In contrast to that, we used the extremely pathogenic and virulent *P. aeruginosa* strain (PA57) that was isolated from the patient with lethal form of CF. It makes a big advantage of our experimental model over the others investigating a usefulness of trained macrophages in a therapy of lung CF. Namely, to avoid PA57 - dependent adverse polarization of macrophages into the BAM phenotype [4], we primed/trained murine macrophages with β-glucan derived from *S. cerevisiae*, the yeast strain showing antimicrobial, anti-inflammatory and antioxidant activities [11]. A quantitative proteomic approach, as well as analysis of secretory profile of inflammatory mediators showed the specific phenotype of the in vitro BG-trained macrophages with the mixture of M1 and M2 markers, as it is described in the Results. Such broad and complex investigation of proteome, secretory properties, expression of phenotypic markers and antimicrobial properties of BG-trained macrophages exposed to *P. aeruginosa* was performed for the first time. Importantly, as evidenced by our in-depth proteomic analysis, BG induced specific wide range of proteins, including proteins related to amelioration of macrophage detrimental effects and to resolution of inflammatory response (Supplementary Tables 1 and 2). Distinct effects from those has been observed with Candida-derived beta-glucans [30]. In our experimental system some of the identified proteins are serpinB2 (8.75-fold increase), antileukoproteinase (SLPI– 11.05-fold increase) and manganese mitochondrial superoxide dismutase (SOD2–7.02-fold increase). In more detail, SerpinB2 (PAI-2, plasminogen activator inhibitor type 2) is substantially up-regulated under multiple inflammatory conditions. It is critical to macrophage survival, promotes a resolution of inflammation and contributes in tissue repair. SerpinB2 deficient mice exhibit impaired responses to infections [31]. Antileukoproteinase (SLPI -secretory leukocyte peptidase inhibitor) is a defense molecule against invading microorganisms. It also protects the mucosal epithelia against inflammatory damage by inhibiting proteolytic enzymes [32]. MnSOD is the mitochondrial antioxidant enzyme acting as the major ROS scavenging enzyme in the cell, protecting a host from adverse effects of oxidative stress [33]. Simultaneously, along with the increase of these proteins, BG decreased macrophage metalloproteinase (MMP12–2.87-fold decrease), an inflammatory enzyme responsible for the host tissue damage belonging to the family of proteins implicated in the pathogenesis of CF lung disease [34].

The above-described changes (these changes in the proteome of) in BG-trained macrophages suggest that such primed macrophages may control the magnitude of inflammation induced by *P. aeruginosa* infections. Nevertheless, it is necessary to explain whether these macrophages are able to kill *P. aeruginosa*. The results from our in vitro experimental studies clearly showed that BG-macrophages effectively phagocytosed and killed PA57 bacteria cells, comparably to naïve macrophages, in spite of the profound reduction of NO synthesis, the key microbicidal molecule [35]. It may be explained by the redundancy of innate immune mechanisms – one of the primary hallmarks of the immune system [36]. Deficit of NO could have been compensated by the overexpression of antibacterial proteins such as SAA-3 (7.78 fold increase) and neutrophil gelatinase–associated lipocalin (NGAL – 4.33-fold increase). Macrophage intracellular SAA-3 is an acute phase protein and innate immune opsonin for Gram-negative bacteria, including *P. aeruginosa* [37]. On the other hand, NGAL is a bacteriostatic glycoprotein due to its ability to capture and to deplete iron-laden bacterial siderophores [38].

Importantly, we have identified quantitative changes in the expression of several proteins involved in the potentiation of phagosome function. The TLR2 receptor, along with its coreceptors CD14 and C-type lectin domain family 7 member A (Clec7a) were all induced in BG-macrophages upon contact with PA57 bacteria cells (2.58, 2.78 and 2.76 fold increase, respectively). Likewise, the Fc-gamma RIII receptor (CD16) which promotes macrophage-mediated phagocytosis [39] was also upregulated (3.05 increase). These changes were accompanied by the 1.64 fold upregulation of V-type proton ATPase subunit S1 (Atp6ap1) – a member of the V-type ATPase complex that mediates the acidification of intracellular organelles [40]. Interestingly, thrombospondin 1 was induced almost 2 fold in activated BG-macrophages. This multifunctional glycoprotein, among many activities, negatively modulates dendritic cell activation and cytokine release, as part of an autocrine feedback loop, contributing to the resolution of inflammation and immune homeostasis [41]. Taken together, proteome signature of PA57-activated BG-macrophages suggests that these cells respond in an orchestrated manner to maintain phagocytic activity despite the reduction in NO synthesis.

The suppression of NO synthesis by BG-trained macrophages restimulated with PA57 is in agreement with other studies. For example, it has been shown that BG from S. cerevisiae inhibited LPS-stimulated NO production in RAW264.7 macrophages [42]. Moreover, in our experimental system this effect was associated with the strong enhancement of TGF-β expression and concomitant profound reduction of NOS2. Previously, it has been well documented that the production of TGF-β serves as a counterbalance for NOS2 suppression [43].

Moreover, BG training of macrophages, apart from changing intracellular protein expression, also markedly affected eicosanoids synthesis, mainly PGE2 and PGD2 prostaglandins with opposite functions. Namely, BG not only increased the concentration of prostaglandin E2 synthase in naïve macrophages (correlated with PGD2 and CD69 repression), but also strongly enhanced its biosynthesis in BG-macrophages restimulated with PA57. It indicates the additive stimulatory effect of BG and PA57 on the expression of PGE2 synthase, in spite of stimulation of distinct receptors, Dectin-1 and TLR-4, respectively. The final effect of PGE2 activity, pro- or anti-inflammatory, will depend on expression of PGE2 receptors (EP1 – EP4). For example, PGE2-EP4-cAMP pathway is associated with anti-inflammatory M2 polarization of macrophages, while stimulation of EP3 receptors on mastocytes induces acute inflammation [44, 45]. On the other hand, the repression of CD69 lectin is undoubtedly a favorable result as CD69 plays an important role in the progression of lung injury. In monocytes/macrophages it has been functionally linked to the 5-lipoxygenase pathway in which the leukotrienes, potent inflammatory mediators, are produced [46].

Concluding, the results of in vitro macrophage training with BG showed that *S. cerevisiae* β-glucan improved the macrophage response to PA57, suggesting its beneficial role in treatment of *P. aeruginosa* infections. Indeed, our in vivo investigations of a role of trained macrophages in the air pouch model of PA57 infection in mice confirmed this hypothesis.

In our opinion, the most important finding of this studies is the profound effect of in vivo trained macrophages on the course of PA57 infection in mice. Transfer of BG trained macrophages not only reduced the local proliferation of bacteria but also suppressed the formation of massive biofilm. Importantly, significantly lower level of serum SAA and reduced number of PA57 CFU in spleen of mice which received trained macrophages indicate their anti-inflammatory properties and ability to inhibit/eliminate bacteremia.

### Final conclusions

Our study demonstrated that training of murine macrophages with *S. cerevisiae* β-glucan, prior to the contact with *P. aeruginosa*, improves macrophage defence properties along with inhibition of secretion of some detrimental inflammatory agents. Therefore, it protects macrophages from the polarization into BAMs, hyperinflammatory phagocytes contributing to the tissue injury at a site of chronic inflammation. Importantly, our study does not exclude the ability of other fungal agents (e.g. chitin) to induce effective innate trained immunity in the treatment of various bacterial infections. Furthermore, we do not favour this derivative over the others but we reveal how BG-stimulation is able to reconcile the expected defence and pro-resolution properties of macrophages, the properties so important in amelioration of recurrent, infectious inflammations. Therefore, we think that training of macrophages with similar β-glucans might be a new therapeutic strategy in biofilm forming *P. aeruginosa* infections, especially lung cystic fibrosis. However, further studies are necessary to examine other routs of β-glucan administration (e.g. *per os*) enabling induction of the trained immunity in humans. Moreover, a better understanding of the mechanisms and consequences of acquired innate immunity is necessary.

### Electronic supplementary material

Below is the link to the electronic supplementary material.

Supplementary Material 1

Supplementary Material 2

Supplementary Material 3

Supplementary Material 4

Supplementary Material 5

Supplementary Material 6

Supplementary Material 7

Supplementary Material 8

Supplementary Material 9

## Data Availability

All data generated and analysed during this study are included in this article. Further inquiries can be directed to the corresponding author.
