# Supplementary figures and images for: Saccharomyces cerevisiae β-glucan improves the response of trained macrophages to severe P. aeruginosa infections
Source: Inflamm Res. 2024 Jun 8;73(8):1283–97. doi: 10.1007/s00011-024-01898-1 (PMC11282130; doi:10.1007/s00011-024-01898-1)

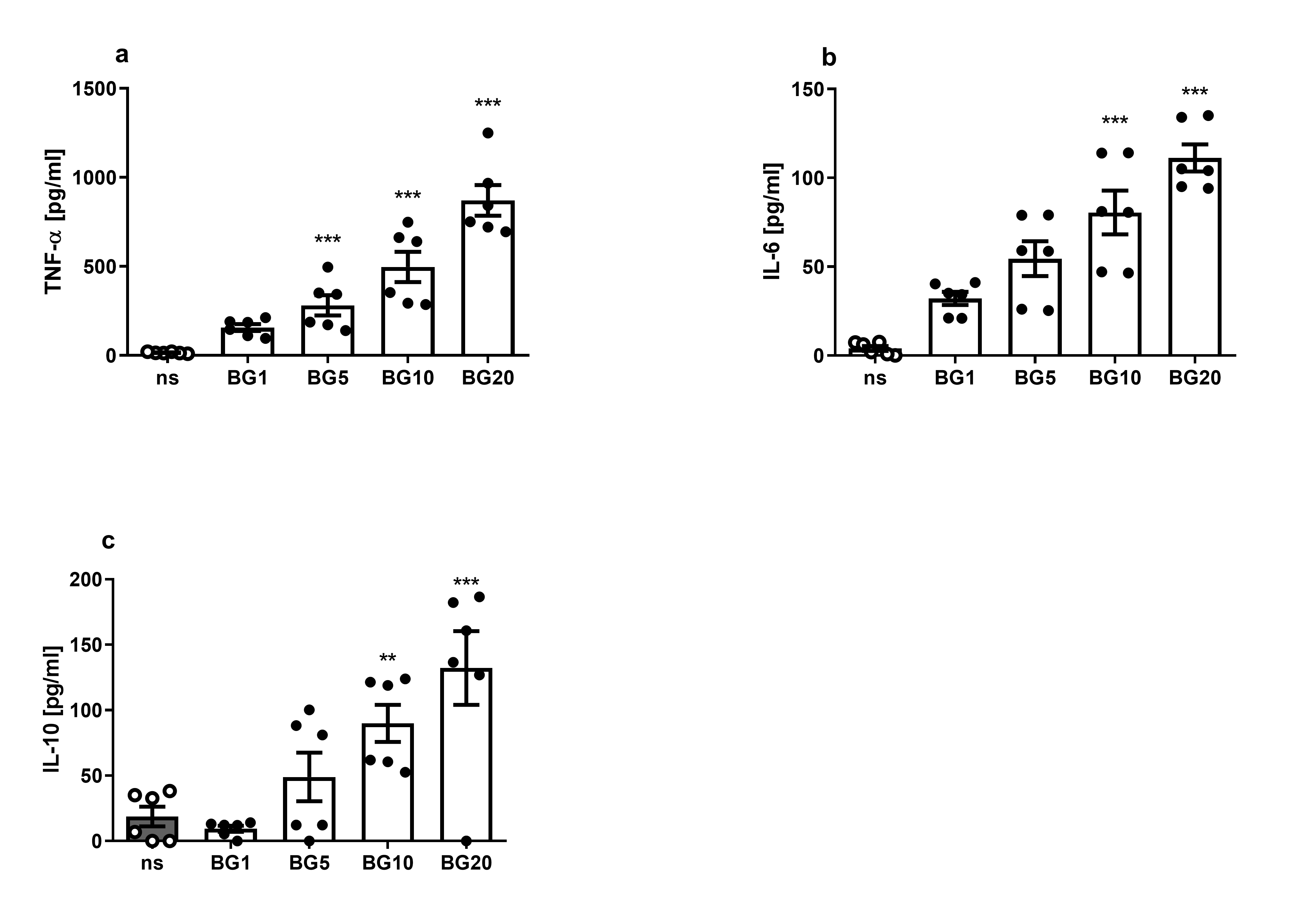

Supplement: Supplementary file 1 — Supplementary Material 1 [file 11_2024_1898_MOESM1_ESM.tif]

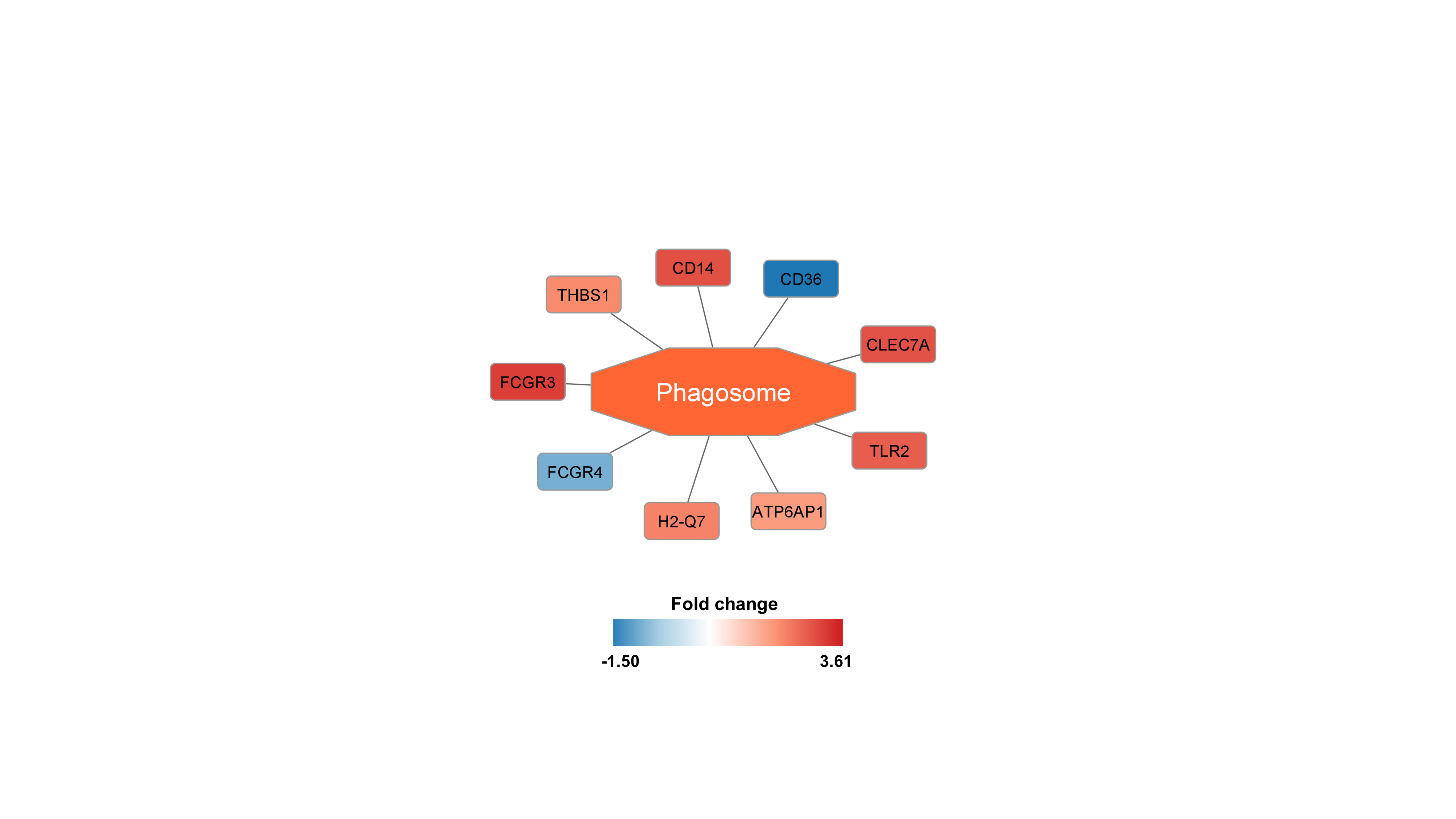

Supplement: Supplementary file 2 — Supplementary Material 2 [file 11_2024_1898_MOESM2_ESM.tif]

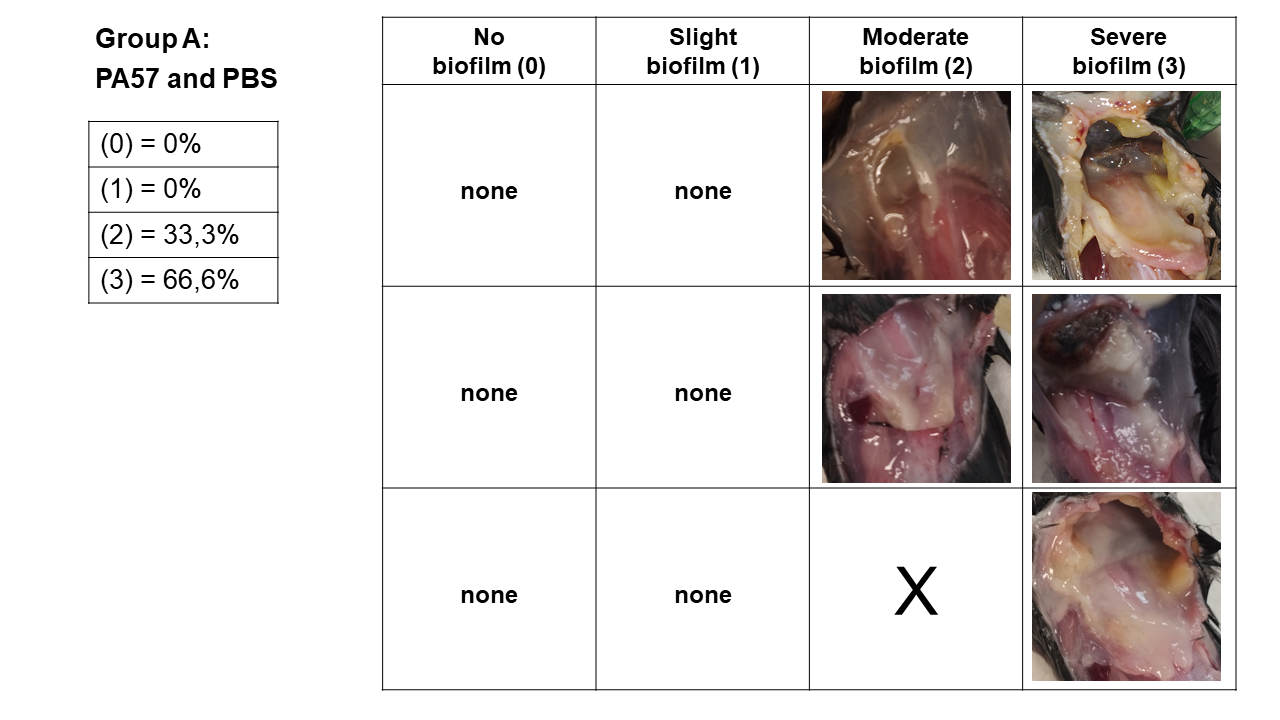

Supplement: Supplementary file 4 — Supplementary Material 4 [file 11_2024_1898_MOESM4_ESM.tif]

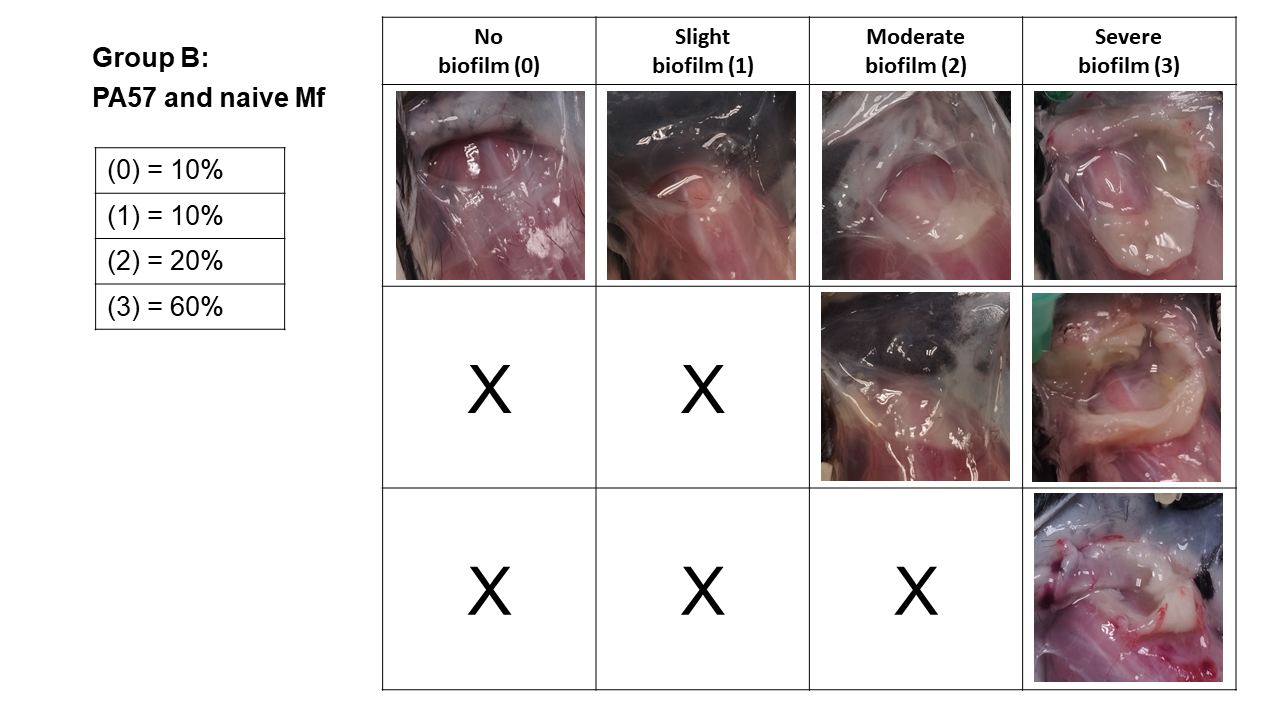

Supplement: Supplementary file 5 — Supplementary Material 5 [file 11_2024_1898_MOESM5_ESM.tif]

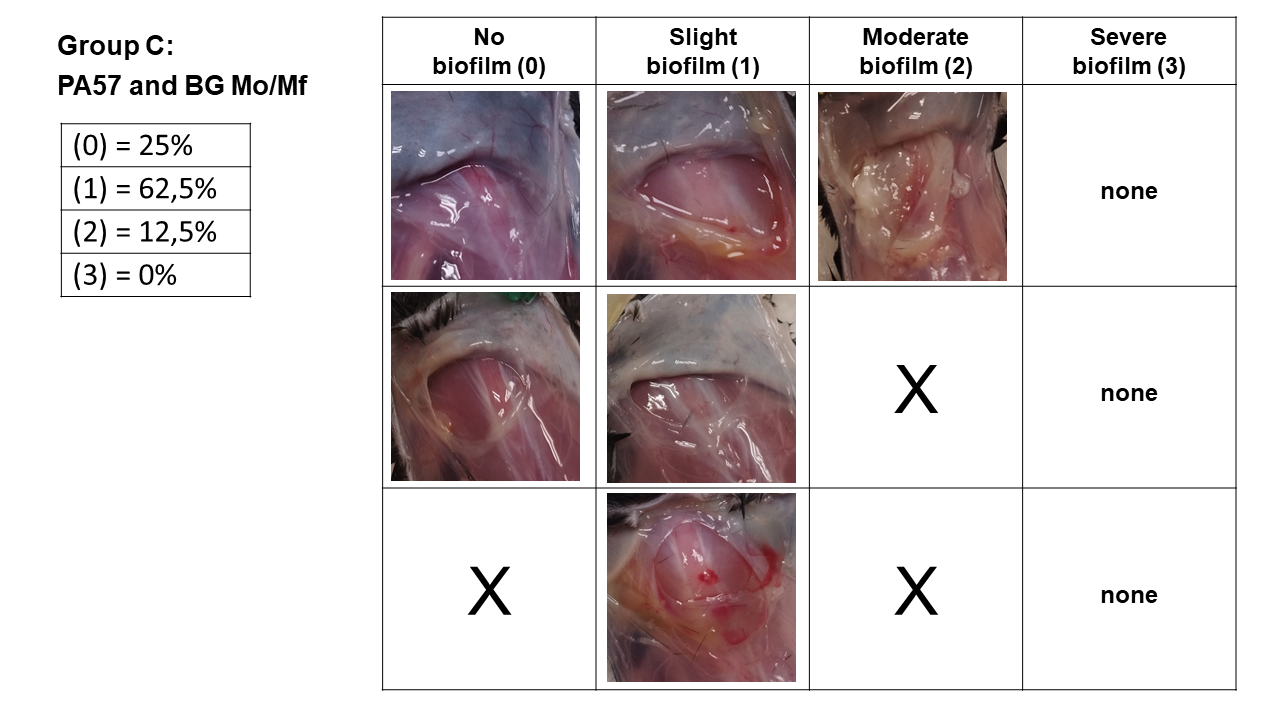

Supplement: Supplementary file 6 — Supplementary Material 6 [file 11_2024_1898_MOESM6_ESM.tif]
